# Impact of Diagonal Branch Angle on Cardiac Outcomes After Sequential Coronary Bypass Surgery

**DOI:** 10.1093/icvts/ivag101

**Published:** 2026-04-15

**Authors:** Akinobu Ohtani, Shin Yajima, Daisuke Yoshioka, Ai Kawamura, Yusuke Misumi, Takuji Kawamura, Shinya Tajima, Masaru Matsuda, Shunsuke Saito, Kazuo Shimamura, Shigeru Miyagawa

**Affiliations:** Department of Cardiovascular Surgery, The University of Osaka, Suita, Osaka 565-0871, Japan; Department of Cardiovascular Surgery, The University of Osaka, Suita, Osaka 565-0871, Japan; Department of Cardiovascular Surgery, The University of Osaka, Suita, Osaka 565-0871, Japan; Department of Cardiovascular Surgery, The University of Osaka, Suita, Osaka 565-0871, Japan; Department of Cardiovascular Surgery, The University of Osaka, Suita, Osaka 565-0871, Japan; Department of Cardiovascular Surgery, The University of Osaka, Suita, Osaka 565-0871, Japan; Department of Cardiovascular Surgery, The University of Osaka, Suita, Osaka 565-0871, Japan; Department of Cardiovascular Surgery, The University of Osaka, Suita, Osaka 565-0871, Japan; Department of Cardiovascular Surgery, The University of Osaka, Suita, Osaka 565-0871, Japan; Department of Cardiovascular Surgery, The University of Osaka, Suita, Osaka 565-0871, Japan; Department of Cardiovascular Surgery, The University of Osaka, Suita, Osaka 565-0871, Japan

**Keywords:** narrow angle configuration, coronary artery bypass, sequential bypass, cardiac event, re-intervention

## Abstract

**Objectives:**

To evaluate whether the diagonal branch anastomotic angle (D-angle) influences outcomes following coronary artery bypass grafting (CABG).

**Methods:**

This study retrospectively examined 197 patients who underwent isolated CABG between 2010 and 2023 using left internal thoracic artery (LITA)-left anterior descending artery (LAD) grafting and clockwise right ITA (RITA)-radial artery (RA) I-composite sequential grafts. Patients were categorized into Narrow (D-angle ≤ 90°, *n* = 16) and Wide (D-angle > 90° or without diagonal anastomosis, *n* = 181) groups.

**Results:**

Mean age was 69 ± 10 and 65 ± 10 years in the Narrow and Wide groups, respectively (*P = *.107). Male sex (93.8% vs 91.2%), diabetes (68.8% vs 57.5%), 3-vessel disease (75.0% vs 85.1%), and off-pump CABG (68.8% vs 76.2%) values were similar; however, the Narrow group had more anastomoses (4.2 ± 0.8 vs 3.4 ± 0.7, *P *< .001). Median follow-up was 5.7 years [3.2-9.1]. Rates of all-cause mortality and adverse cardiac events did not differ significantly (*P = *.193; *P = *.074). However, freedom from adverse sequential graft events was lower in the Narrow group, with 1-, 5-, and 10-year estimates of 74.0%, 74.0%, and 74.0%, compared with 93.6%, 92.0%, and 92.0% in the Wide group, respectively (log-rank *P = *.008). Narrow D-angle remained an independent predictor in multivariate analysis (adjusted hazard ratio, 4.3; 95% confidence interval, 1.3-13.7; *P = *.014).

**Conclusions:**

A narrow D-angle was independently associated with increased incidence of adverse sequential graft events.

## Graphical abstract

**Figure:**
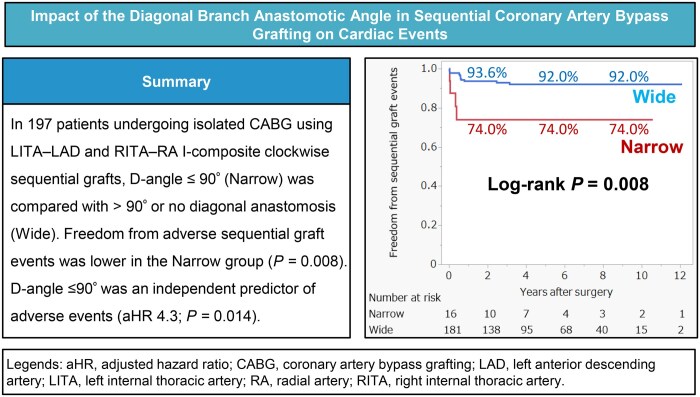

## INTRODUCTION

Coronary artery bypass grafting (CABG) remains central to myocardial revascularization. Contemporary guidelines recommend *in situ* left internal thoracic artery (LITA)-left anterior descending artery (LAD) grafting as the default strategy and advocate broader arterial use. For non-LAD targets, the radial artery (RA) is preferred over the saphenous vein graft (SVG).^1^^,^^2^ However, beyond the LITA-LAD graft, no consensus exists regarding the optimal second conduit (eg, sequential/composite strategies), and clinical practice varies widely.^3^

Long-term evidence indicates superior survival with multiple or total arterial strategies compared with those including SVGs. Pooled data further support RA over SVG as the second conduit.^4^ However, these findings provide limited guidance for configuration-specific decisions in multi-anastomotic constructs.

The right ITA (RITA)-RA I-composite graft enables an aorta no-touch approach, frequent off-pump techniques, and all-arterial revascularization of the lateral wall through sequential bypasses. Sequential RA grafting is a well-established technique associated with excellent long-term patency rates.^5^ The ITA-RA I-composite graft achieves long-term outcomes superior to those of a free RA graft but comparable to those of ITA alone.^6^ Its application to severely stenotic targets with clockwise sequential anastomoses enhances mid-term patency.^7^ In total arterial revascularization, bilateral ITA with an RA I-composite is associated with improved survival compared with single ITA with an RA Y-composite,^8^ supporting the validity of this approach.

In sequential diagonal revascularization, the RA graft may acutely deflect at the diagonal anastomosis. Although sequential grafting is well-established, configuration-specific clinical thresholds^1^ and the impact of the D-angle remain undefined.

We therefore evaluated whether the angle between graft segments before and after the diagonal branch anastomosis is associated with patient and graft outcomes following CABG.

## METHODS

### Ethical statement

This study was conducted following the Declaration of Helsinki and the World Medical Association Declaration of Taipei. The institutional ethics committee of Osaka University Hospital approved and monitors the establishment and ongoing use of the Osaka Cardiovascular Research Group database. The committee approved this retrospective protocol and waived written informed consent (Reference no. 08218-12; March 25, 2025).

### Study cohort

The patient selection flow diagram is shown in **Figure S1**. This single-centre retrospective study included all patients undergoing primary isolated CABG at our institution between January 2010 and June 2023. Among 1123 consecutive cases, 231 patients received CABG with a LITA-LAD graft and a clockwise I-composite graft using RITA-RA (Y-composite grafts were not included) to the diagonal, left circumflex, and/or right coronary artery (RCA) territories. Clockwise configuration refers to the sequential routing of the RITA-RA I-composite graft, in which flow proceeds from the more proximal anastomosis (diagonal branch, if present) towards the lateral wall (circumflex territory) and potentially to the posterior/inferior wall. In the anterior view of the heart, this routing follows a clockwise direction. Patients with counterclockwise I-composite sequential anastomoses (ie, RCA-first routing; *n* = 27), dual-inflow bypass to the RCA (*n* = 2), interposed SVG (*n* = 1), and transverse sinus routing of the RITA (*n* = 1), as well as those without postoperative coronary computed tomography angiography (CCTA), were excluded. The final cohort comprised 197 patients.

### Definition of the D-angle

**Figure 1.**
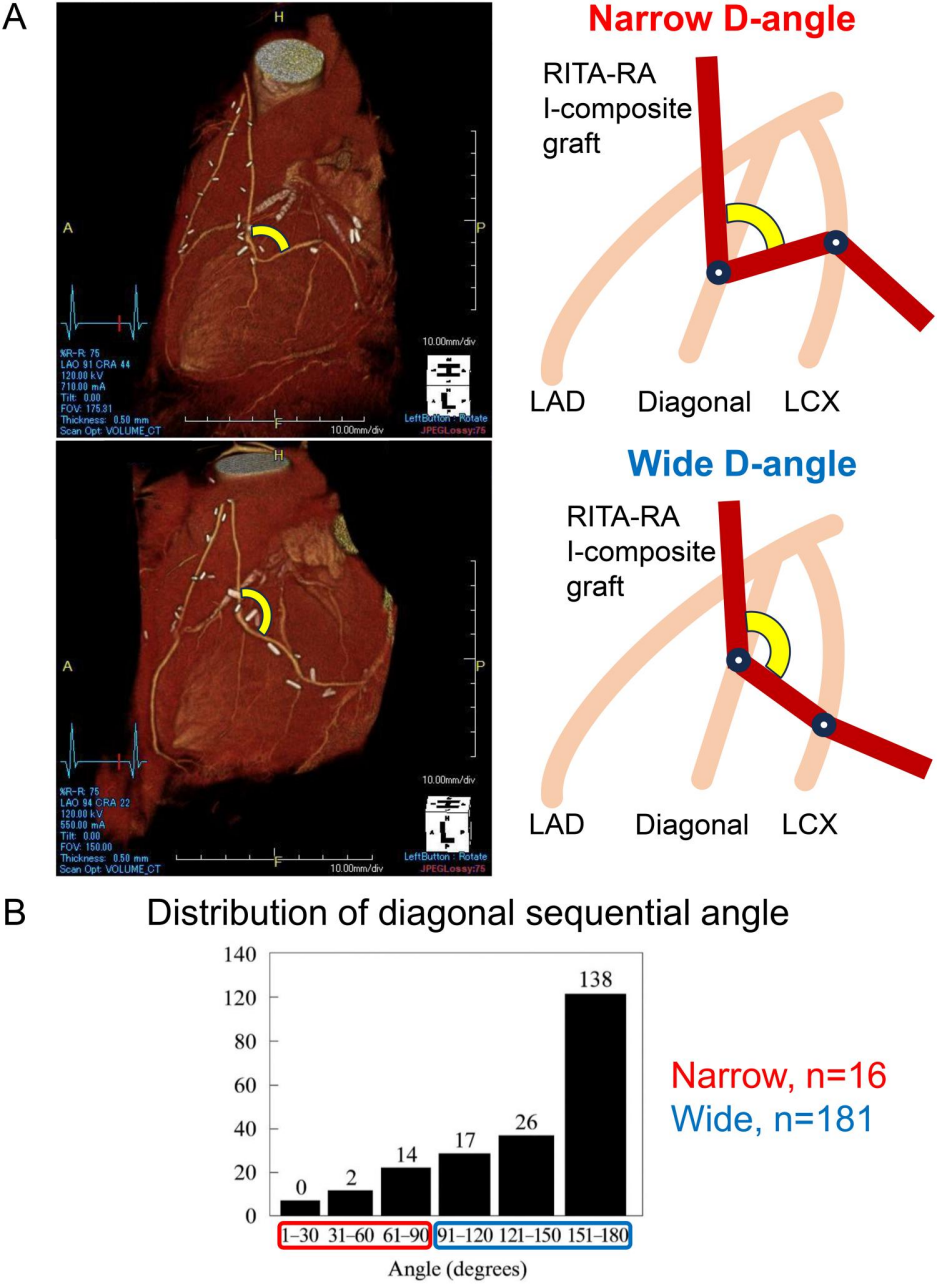
Diagonal Sequential Angle. (A) Schematic representation of the diagonal sequential angle in the Narrow and Wide groups. The D-angle is defined as the angle between the proximal and distal graft segments at the diagonal branch anastomosis. (B) Distribution of diagonal sequential angle. Abbreviations: LAD, left anterior descending artery; LCX, left circumflex; RA, radial artery; RITA, right internal thoracic artery

The D-angle was defined as the smaller angle between graft segments before and after the diagonal branch anastomosis (**Figure 1A**). Three-dimensional CCTA datasets were reconstructed, and the D-angle was measured on AquariusNET Viewer, version 4.4.14.P1 (TeraRecon, Durham, NC, USA). All measurements were reviewed and confirmed by 9 cardiovascular surgeons. A total of 16 patients were classified into the Narrow group (D-angle ≤ 90°) and 181 into the Wide group (D-angle > 90° or patients without a diagonal branch). This 90° cutoff was adopted based on haemodynamic principles regarding pressure loss at rectangular bends^9^^,^^10^ and its clinical utility as a clear intraoperative landmark. For sequential bypass grafts without a diagonal branch anastomosis, the D-angle was defined as 180° (**Figure 1B**).

### Intraoperative measurements

Intraoperative graft flow was assessed using transit-time flowmetry for both the LITA and RITA-RA composite grafts prior to chest closure under stable haemodynamic conditions. The diameter of the target coronary arteries was estimated intraoperatively by referencing the size of the intracoronary shunts inserted during anastomosis.

### Study end-points

The study end-points were all-cause mortality and adverse cardiac or sequential graft events. Adverse cardiac events were defined as cardiac death, myocardial infarction, or coronary reintervention (patient-level; any territory). Adverse sequential graft events comprised myocardial infarction or reintervention attributable to the distal sequential graft beyond the diagonal anastomosis, plus occlusion of that distal segment (graft-specific; excludes cardiac death).

### Follow-up

Following hospital discharge, patients attended outpatient visits at 1, 3, 6, and 12 months, and annually thereafter. Follow-up data were obtained from medical records and, when necessary, supplemented by telephone or postal correspondence. The median follow-up duration was 5.7 years (interquartile range [IQR], 3.2-9.1 years).

### Statistical analysis

Continuous variables are presented as mean ± standard deviation (SD) or median (IQR). Group comparisons were performed using χ^2^ or Fisher’s exact test for categorical variables and the Student’s t-test, Mann-Whitney U-test, or paired t-test for continuous variables. The Cox proportional hazards model was used for univariate and multivariate analyses to assess the association between D-angle and adverse sequential graft events. Overall survival and freedom from adverse cardiac or sequential graft events were analysed using the Kaplan-Meier method, with differences assessed by the log-rank test. Variables with *P *< .10 in the univariate analysis of adverse sequential graft events were entered into a multivariate Cox model. A *P* value < .05 was considered statistically significant. Statistical analyses were conducted using JMP Pro 17 (SAS Institute, Cary, NC, United States).

## RESULTS

### Preoperative patient characteristics and operative results

Baseline patient characteristics are summarized in **Table 1**. In the Narrow and Wide groups, the mean age was 69 ± 10 vs 65 ± 10 years (*P* = .107), male proportion was 93.8% vs 91.2% (*P* = 1.000), diabetes mellitus prevalence was 68.8% vs 57.5% (*P* = .438), current smoking prevalence was 25.0% vs 13.8% (*P* = .263), 3-vessel disease incidence was 75.0% vs 85.1% (*P* = .287), left main disease incidence was 50.0% vs 37.0% (*P* = .421), and left ventricular ejection fraction was 55 ± 12% vs 55 ± 14% (*P* = .855), respectively. All baseline variables were statistically comparable between the 2 groups.

**Table 1.** Baseline Characteristics

Characteristic	Total (*N* = 197)	Narrow (*N* = 16)	Wide (*N* = 181)	*P* value
Age (years)	65 ± 10	69 ± 10	65 ± 10	.107
Male sex (*n*, %)	180 (91.4)	15 (93.8)	165 (91.2)	1.000
BMI (kg/m^2^)	23.8 ± 3.5	22.6 ± 2.7	23.9 ± 3.6	.157
Current smoker (*n*, %)	29 (14.7)	4 (25.0)	25 (13.8)	.263
Hypertension (*n*, %)	139 (70.9)	12 (75.0)	127 (70.2)	.782
Diabetes mellitus (*n*, %)	115 (58.4)	11 (68.8)	104 (57.5)	.438
Dyslipidaemia (*n*, %)	161 (82.1)	13 (81.3)	148 (81.8)	.952
eGFR (mL/min/1.73m^2^)	67.4 ± 19.1	63.3 ± 12.7	67.7 ± 19.5	.375
History of myocardial infarction (*n*, %)	6 (3.0)	0	6 (3.3)	1.000
Acute	5 (2.5)	0	5 (2.8)	1.000
Chronic	1 (0.5)	0	1 (0.6)	1.000
Coronary disease (*n*, %)				
1-Vessel disease	0	0	0	N/A
2-Vessel disease	31 (15.7)	4 (25.0)	27 (14.9)	.287
3-Vessel disease	166 (84.3)	12 (75.0)	154 (85.1)	.287
Left main disease	75 (38.1)	8 (50.0)	67 (37.0)	.421
History of CVD (*n*, %)	15 (7.6)	3 (18.8)	12 (6.6)	.109
COPD (≥moderate) (*n*, %)	6 (3.0)	0	6 (3.3)	1.000
Atrial fibrillation (*n*, %)	10 (5.1)	1 (6.3)	9 (5.0)	.580
Peripheral vascular disease (*n*, %)	22 (11.2)	2 (12.5)	20 (11.0)	.695
Left ventricular ejection fraction (%)	55 ± 14	55 ± 12	55 ± 14	.855
<40% (*n*, %)	31 (15.7)	3 (18.8)	28 (15.5)	.722
Preoperative IABP (*n*, %)	11 (5.6)	0	11 (6.1)	.589
V-A ECMO (*n*, %)	1 (0.5)	0	1 (0.6)	1.000

Abbreviations: BMI, body mass index; COPD, chronic obstructive pulmonary disease; CVD, cerebrovascular disease; eGFR, estimated glomerular filtration rate; IABP, intra-aortic balloon pump; N/A, not applicable; V-A ECMO, veno-arterial extracorporeal membrane oxygenation.

Operative details are presented in **Table 2**. The rates of emergent surgery (6.3% vs 6.1%, *P *= 1.000), off-pump CABG (68.8% vs 76.2%, *P *= .546), and severe terminal sequential anastomosis stenosis (81.3% vs 92.8%, *P *= .128) were similar in the Narrow and Wide groups. Total anastomoses were significantly higher in the Narrow group (4.2 ± 0.8 vs 3.4 ± 0.7, *P *< .001), driven by additional diagonal branch anastomoses (1.0 ± 0.0 vs 0.3 ± 0.5, *P *< .001). All other operative variables were similar between groups. Intraoperative transit-time flow measurement showed no significant difference in the RITA-RA composite graft flow between the Narrow and Wide groups (49 ± 32 mL/min vs 55 ± 35 mL/min, *P = *.525). Target vessel diameters were generally comparable, with no significant differences observed in the LAD or diagonal branch.

**Table 2.** Operation Details

Variable	Total (*N* = 197)	Narrow (*N* = 16)	Wide (*N* = 181)	*P* value
Surgical acuity (*n*, %)				
Elective	169 (85.8)	15 (93.8)	153 (85.0)	.478
Urgent	16 (8.1)	0	16 (8.9)	.371
Emergent	12 (6.1)	1 (6.3)	11 (6.1)	1.000
No. of anastomosis (*n*, mean ± SD)				
Total	3.4 ± 0.7	4.2 ± 0.8	3.4 ± 0.7	**<.001**
Left anterior descending artery	1.0 ± 0.1	1.0 ± 0.0	1.0 ± 0.1	.675
Diagonal branch	0.3 ± 0.5	1.0 ± 0.0	0.3 ± 0.5	**<.001**
Left circumflex	1.2 ± 0.5	1.3 ± 0.4	1.2 ± 0.5	.961
Right coronary artery	0.8 ± 0.6	1.0 ± 0.6	0.8 ± 0.6	.257
Target vessel diameter (mm, mean ± SD)				
Left anterior descending artery	1.5 ± 0.2	1.4 ± 0.3	1.5 ± 0.2	.247
Diagonal branch	1.3 ± 0.2	1.2 ± 0.2	1.3 ± 0.2	.235
Left circumflex	1.3 ± 0.2	1.1 ± 0.2	1.3 ± 0.2	.012
Right coronary artery	1.3 ± 0.2	1.2 ± 0.2	1.3 ± 0.2	.081
Severe stenosis (≥90%) of the terminal sequential anastomosis (*n*, %)	181 (91.9)	13 (81.3)	168 (92.8)	.128
Operation time (min, mean ± SD)	316 ± 70	333 ± 74	315 ± 70	.313
Cardiopulmonary bypass status (*n*, %)				
Off-pump	149 (75.6)	11 (68.8)	138 (76.2)	.546
On-pump beating	48 (24.4)	5 (31.3)	43 (23.8)	.546
Cardiac arrest	0	0	0	N/A
Intraoperative Transit-time Flow (mL/min, mean ± SD)				
LITA-LAD graft	46 ± 29	36 ± 15	47 ± 30	.157
RITA-RA I-composite sequential graft	55 ± 34	49 ± 32	55 ± 35	.525

Abbreviations: LAD, left anterior descending artery; LITA, left internal thoracic artery; N/A, not applicable; RA, radial artery; RITA, right internal thoracic artery; SD, standard deviation. Bold values indicate statistical significance (*P* < .05).

### Overall survival and cardiac events

In the Narrow and Wide groups, early results showed a 30-day operative mortality of 0%, while adverse cardiac events occurred in 0% vs 1.1% (*P* = 1.000) and cerebrovascular accidents in 0% vs 1.1% (*P* = 1.000) of patients. Adverse sequential graft events were more frequent in the Narrow group (12.5% vs 2.2%, *P = *.077). All other early postoperative outcomes were comparable (**Table 3**).

**Table 3.** Operative Outcomes

Variable	Total (*N* = 197)	Narrow (*N* = 16)	Wide (*N* = 181)	*P* value
Operative mortality (*n*, %)	0	0	0	N/A
Adverse cardiac events (*n*, %)	2 (1.0)	0	2 (1.1)	1.000
Cardiac death	0	0	0	N/A
Myocardial infarction	0	0	0	N/A
Re-intervention	2 (1.0)	0	2 (1.1)	1.000
Cerebrovascular accidents (*n*, %)	2 (1.0)	0	2 (1.1)	1.000
Adverse sequential graft events (*n*, %)	6 (3.0)	2 (12.5)	4 (2.2)	.077
Long-term follow-up				
Mortality (*n*, %)	12 (6.1)	2 (12.5)	10 (5.5)	.253
Adverse cardiac events (*n*, %)	26 (13.2)	4 (25.0)	22 (12.2)	.149
Cardiac death	3 (1.5)	0	3 (1.7)	1.000
Myocardial infarction	3 (1.5)	0	3 (1.7)	1.000
Re-intervention	24 (12.2)	4 (25.0)	20 (11.0)	.113
Cerebrovascular accidents (*n*, %)	6 (3.0)	0	6 (3.3)	1.000
Adverse sequential graft events (*n*, %)	17 (8.6)	4 (25.0)	13 (7.2)	**.036**

Abbreviation: N/A, not applicable. Bold values indicate statistical significance (*P* < .05).

**Figure 2.**
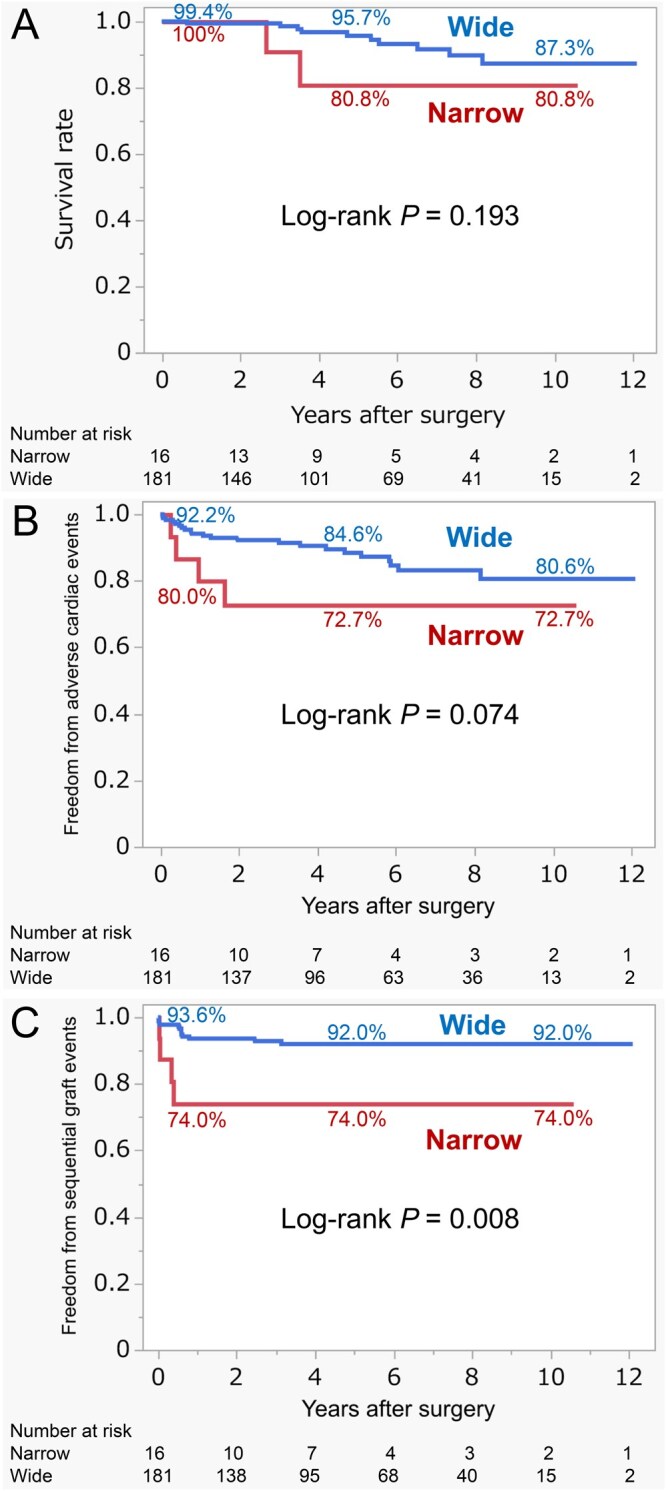
Kaplan-Meier Analysis of Clinical Outcomes. (A) Survival rate, (B) freedom from adverse cardiac events, and (C) freedom from adverse sequential graft events. No significant differences were observed in survival rate or adverse cardiac events between the Narrow and Wide groups. Freedom from adverse sequential graft events was lower in the Narrow group compared with the Wide group

At long-term follow-up, Kaplan-Meier-estimated overall survival at 1, 5, and 10 years was 100%, 80.8 ± 12.3%, and 80.8 ± 12.3% in the Narrow group, compared with 99.4 ± 0.6%, 95.7 ± 1.9%, and 87.3 ± 4.1% in the Wide group, respectively (log-rank *P = *.193; **Figure 2A**). Freedom from adverse cardiac events at 1, 5, and 10 years was 80.0 ± 10.3%, 72.7 ± 11.7%, and 72.7 ± 11.7% in the Narrow group, compared with 94.2 ± 1.8%, 88.4 ± 2.7%, and 80.6 ± 4.4% in the Wide group (log-rank *P = *.074; **Figure 2B**). Freedom from adverse sequential graft events at 1, 5, and 10 years was consistently 74.0 ± 11.2% in the Narrow group, compared to 93.6 ± 1.9%, 92.0 ± 2.2%, and 92.0 ± 2.2% in the Wide group, respectively (log-rank *P = *.008; **Figure 2C**). In univariate analysis, a narrow D-angle was associated with a significantly increased risk of adverse sequential graft events compared with a wide D-angle (hazard ratio [HR], 4.1; 95% confidence interval [CI], 1.3-12.5; *P = *.014). D-angle ≤90° remained an independent predictor in the multivariate analysis (adjusted HR, 4.3; 95% CI, 1.3-13.7; *P = *.014; **Table 4**).

**Table 4.** Risk Analysis for Adverse Sequential Graft Events: Cox Proportional Hazards Regression Analysis

Variable	Univariate analysis		Multivariate analysis	
	Crude HR (95% CI)	*P* value	Adjusted HR (95% CI)	*P* value
Narrow diagonal sequential angle	4.1 (1.3-12.5)	**.014**	4.3 (1.3-13.7)	**.014**
Age	1.0 (1.0-1.1)	.718	1.0 (1.0-1.1)	.873
Male sex	0.3 (0.1-0.8)	**.021**	0.3 (0.1-0.8)	**.017**
BMI (kg/m^2^)	1.0 (0.9-1.1)	.866		
Hypertension	2.1 (0.6-7.3)	.249		
Diabetes mellitus	2.6 (0.8-7.9)	.099	2.7 (0.9-8.3)	.088
Dyslipidaemia	1.6 (0.4-6.8)	.560		
eGFR (mL/min/1.73m^2^)	1.0 (1.0-1.0)	.188		
Prior myocardial infarction	0.0	.999		
2-Vessel disease	0.7 (0.2-2.9)	.593		
3-Vessel disease	1.5 (0.3-6.5)	.593		
Left main disease	1.1 (0.4-3.0)	.795		
History of CVD	1.8 (0.4-7.7)	.455		
Left ventricular ejection fraction (%)	1.0 (1.0-1.1)	.316		
Low EF (< 40%)	0.3 (0.0-2.3)	.257		
Preoperative IABP	1.0 (0.1-7.7)	.986		
V-A ECMO	0.0	.999		
Elective	1.2 (0.3-5.2)	.811		
Urgent	0.7 (0.1-5.3)	.727		
Emergent	1.1 (0.1-8.0)	.951		
No. of anastomosis				
Total	1.2 (0.6-2.2)	.630		
Left anterior descending artery	0.0	.999		
Diagonal branch	1.0 (0.4-2.6)	.960		
Left circumflex	0.9 (0.4-2.2)	.853		
Right coronary artery	1.4 (0.6-3.3)	.420		
Severe stenosis (≥90%) of the terminal sequential anastomosis	0.4 (0.1-1.4)	.141		
Operation time	1.0 (1.0-1.0)	.995		
Off-pump	2.5 (0.6-11.1)	.215		

Abbreviations: BMI, body mass index; CI, confidence interval; CVD, cerebrovascular disease; EF, ejection fraction; eGFR, estimated glomerular filtration rate; HR, hazard ratio; IABP, intra-aortic balloon pump; V-A ECMO, veno-arterial extracorporeal membrane oxygenation. Bold values indicate statistical significance (*P* < .05).

**Figure 3.**
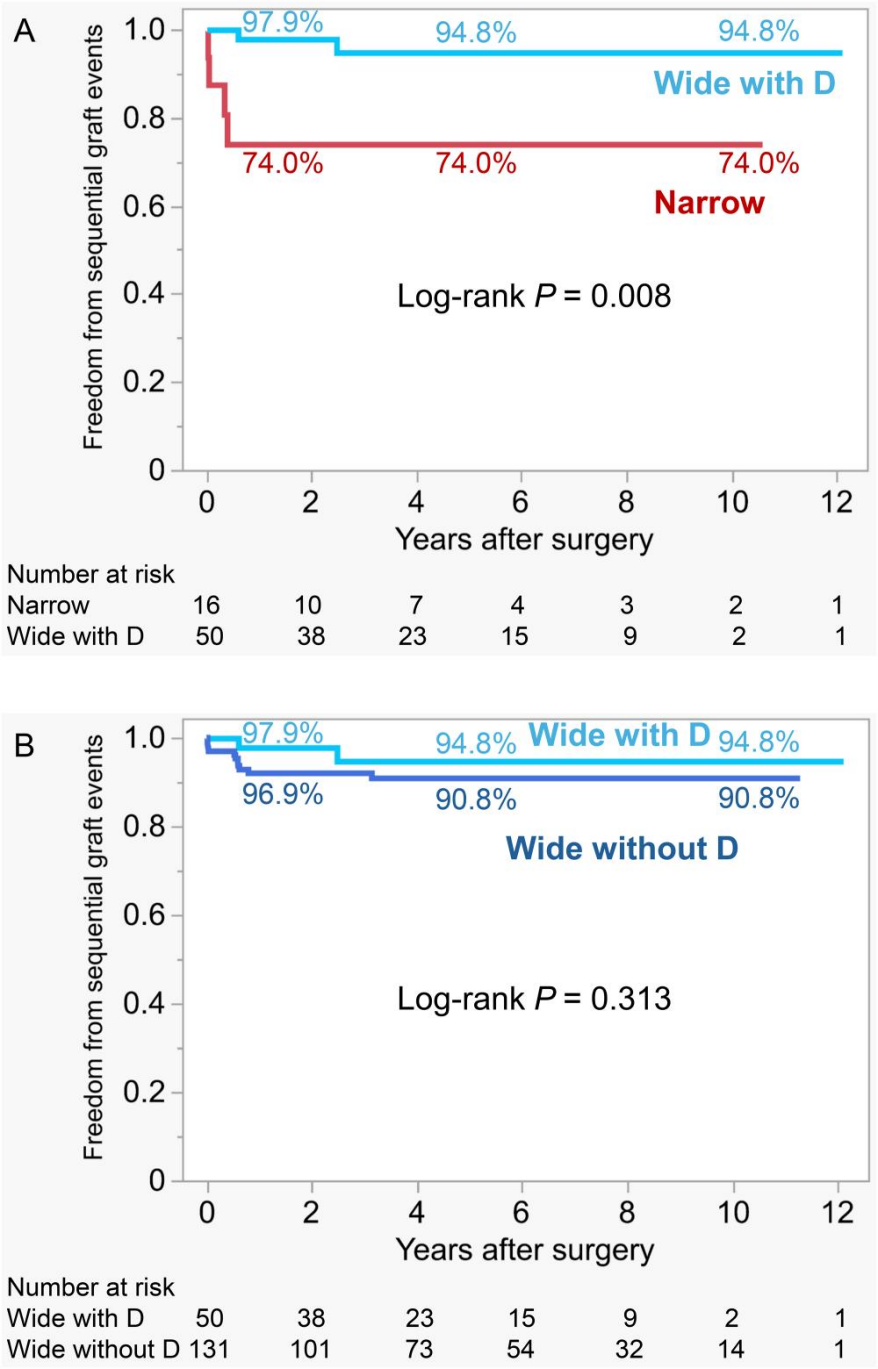
Stratified Analysis of Adverse Sequential Graft Events by Diagonal Branch Anastomosis. (A) Analysis restricted to patients with a diagonal branch anastomosis. Kaplan-Meier estimates compare the Narrow group (*n* = 16) with the Wide subgroup with a diagonal anastomosis (Wide with D, *n* = 50). The Narrow group demonstrated a significantly higher incidence of adverse sequential graft events compared with the Wide with D subgroup (log-rank *P =* .009). (B) Comparison within the Wide group stratified by the presence of a diagonal branch anastomosis. Wide with D, Wide group with a diagonal branch anastomosis (*n* = 50); Wide without D, Wide group without a diagonal branch anastomosis (*n* = 131). No significant difference was observed between the 2 subgroups (log-rank *P =* .313)

In stratified analysis to distinguish the impact of the D-angle from the potential confounding effects of the number of anastomoses and the diagonal anastomosis itself, the Narrow group demonstrated a significantly higher incidence of adverse sequential graft events compared with the Wide subgroup with a diagonal anastomosis (log-rank *P = *.009; **Figure 3A**). Conversely, within the Wide group, freedom from adverse sequential graft events did not differ significantly between patients with and without a diagonal branch anastomosis (log-rank *P = *.313; **Figure 3B**). Comparison based on the surgical platform showed no significant difference in freedom from adverse sequential graft events between patients undergoing off-pump and on-pump CABG (**Figure S2**).

## DISCUSSION

Sequential graft geometry has long been recognized as a determinant of patency.^9^^,^^10^ In this study, approximately 10% of patients exhibited a narrow D-angle, which was independently associated with an increased incidence of adverse sequential graft events distal to the diagonal branch, along with a trend towards reduced freedom from adverse cardiac events. The magnitude of the observed effect, with an adjusted HR exceeding 4.0, suggests a potential clinical impact of the narrow D-angle on graft failure, although the wide CI warrants cautious interpretation.

Stratified analysis demonstrated that the Narrow group had significantly worse outcomes compared with the Wide subgroup with a diagonal anastomosis, indicating that the adverse events are attributable to the unfavourable Narrow geometry (**Figure 3A**). Furthermore, within the Wide group, freedom from adverse sequential graft events was comparable between patients with and without a diagonal branch anastomosis (**Figure 3B**). This serves as a critical negative control, demonstrating that the diagonal anastomosis itself is not a risk factor. Collectively, these findings imply that diagonal branch anastomosis can be safely incorporated into sequential grafting strategies without compromising distal patency, provided that a favourable anastomotic angle is secured. Prior studies have not quantified a diagonal-specific postoperative bend or described its population distribution. Therefore, this study introduces the D-angle as a novel, image-derived geometric parameter and demonstrates that acute bending is not uncommon in routine surgical practice. This parameter may be incorporated into preoperative planning and intraoperative routing strategies. Postoperative coronary CT angiography further provides non-invasive visualization of graft course and anastomotic geometry, enabling the assessment of such descriptors and the identification of kinking or acute bends.^11^

Haemodynamic principles offer a mechanistic explanation for these findings.^9^ When flow traverses a short-radius bend of approximately 90°, 2 key effects occur: (1) axial velocity redistributes, with peak velocity shifting towards the outer wall, while a low-velocity separation pocket forms downstream along the inner wall; and (2) a net pressure drop occurs across the bend, characterized by outer-wall pressure elevation, inner-wall pressure reduction, and an overall downstream pressure deficit from momentum redirection and frictional loss. These patterns are well documented in 90° pipe-bend models.^10^

Applied to coronary bypass grafting, a narrow D-angle exposes the subsequent graft segment to unfavourable inflow conditions—specifically, a low wall shear stress zone along the inner wall combined with diminished distal driving pressure. Such conditions may promote early thrombus formation, intimal hyperplasia, and impaired perfusion.^9^ In RA grafts, low or competitive flow may further predispose to vasospasm, manifesting as the string sign and exacerbating luminal narrowing.^12^^,^^13^

Consistent with this mechanism, adverse sequential graft events distal to the diagonal branch clustered predominantly within the first postoperative year, with minimal subsequent accrual thereafter. This temporal pattern implicates early haemodynamic failure, rather than delayed remodelling, drives adverse outcomes with narrow D-angles. Intraoperative graft flow was comparable between groups. Despite more anastomoses in the Narrow group (4.2 ± 0.8 vs 3.4 ± 0.7, *P* < .001), which would be expected to increase flow via a larger distal vascular bed, graft flow was not higher—suggesting a haemodynamic constraint imposed by a narrow D-angle. This suggests that a narrow D-angle may not reduce flow immediately, but may instead generate local flow disturbances, undetectable by standard flowmetry, that may predispose to subacute graft failure.

Cardiac surgeons generally aim to avoid graft kinking and optimize flow through careful intraoperative routing.^14^ In our practice, the graft course is visualized with the heart in its anatomical position, and anastomotic sites are pre-marked to promote smooth curvature. Selective pericardial fixation is occasionally employed to restore a gentle arc when an overly straight segment is observed. Despite these precautions, narrow D-angles may still arise due to anatomical and technical constraints. A discrepancy between the intended straight-graft course and the true diagonal stenosis location can leave excess graft length, creating a V-shaped course, particularly when the diagonal branch anastomosis is distal and the left circumflex anastomosis is proximal. Pericardial stitches may not fully correct the angulation when the graft course is structurally constrained by the relative positions of the anastomotic sites.

Additionally, in RITA-RA I-composite grafts, a diagonal anastomosis placed near the composite junction to extend reach may appear satisfactory during cardiac displacement; however, once the heart is returned to its anatomical position, the RITA side is slightly drawn, producing subtle straightening that further narrows the D-angle.^7^ It is important to note that while sequential grafting to the obtuse marginal branch is a common configuration, the prevalence of obtuse marginal targets was comparable between groups. The narrow angulation can also occur with other targets, such as the posterolateral branch. Furthermore, this angulation can be deceptive; a graft loop may appear satisfactory during cardiac displacement but form a sharp V-shape in the anatomical position. This occult nature underscores the necessity of meticulous preoperative planning. Loss of diagonal branch flow has been associated with adverse outcomes^15^ and achieving complete revascularization contributes to long-term survival.^16^ Accordingly, the RITA-RA I-composite graft facilitates complete revascularization while preserving the advantages of total arterial revascularization. When a narrow D-angle is anticipated because of anatomical constraints, alternative strategies—such as individual grafting or hybrid revascularization for the diagonal branch—may reduce adverse sequential graft events. Notably, our findings indicate that the narrow geometry is often a preventable technical issue. Consideration of the D-angle allows proactive adjustment of graft configuration to secure a favourable geometry or switch to these alternative strategies, thereby ensuring patient safety. Given recent initiatives to promote RA usage in multi-arterial CABG,^17^ surgeons should be vigilant about these geometric pitfalls to maximize the benefit of arterial revascularization.

This study has limitations. First, this was a single-centre retrospective analysis with a relatively small cohort, which may have limited statistical power to detect differences in broader clinical outcomes. Although a formal a priori sample size calculation was not performed, the observed effect size (HR > 4.0) suggests that the current sample size provided sufficient statistical power to detect a significant difference in adverse sequential graft events. However, given the limited sample size, statistical adjustments such as propensity score matching were not feasible. Consequently, our findings should be considered hypothesis-generating. Further validation in larger, multicentre registries employing rigorous adjustments is warranted to generalize these results.

Second, follow-up was incomplete (83.2%), potentially influencing the reliability of long-term findings. Third, D-angle quantification was derived from routine postoperative CCTA without protocol harmonization, leading to inter-scan differences that may have introduced non-differential measurement error. Fourth, although the diameters of the major target vessels (LAD and diagonal branch) were comparable between groups, comprehensive intraoperative sizing data were not universally available, and the potential influence of target vessel size cannot be completely excluded.

## CONCLUSION

Among patients undergoing clockwise RITA-RA I-composite sequential bypass in addition to LITA-LAD grafting, a narrow D-angle, as assessed by CCTA, was independently associated with a significantly elevated risk of adverse sequential graft events distal to the diagonal branch. These findings emphasize the importance of meticulous preoperative planning and careful intraoperative routing to minimize narrow D-angle formation during sequential diagonal reconstruction.

## Supplementary Material

ivag101_Supplementary_Data

## Data Availability

The data underlying this article will be shared on reasonable request to the corresponding author.

## ABBREVIATIONS

CABG: Coronary artery bypass grafting
CCTA: Coronary computed tomography angiography
CI: Confidence interval
HR: Hazard ratio
IQR: Interquartile range
ITA: Internal thoracic artery
LAD: Left anterior descending (coronary) artery
LITA: Left internal thoracic artery
RA: Radial artery
RCA: Right coronary artery
RITA: Right internal thoracic artery
SD: Standard deviation
SVG: Saphenous vein graft
