# Supplementary figures and images for: Impact of Diagonal Branch Angle on Cardiac Outcomes After Sequential Coronary Bypass Surgery
Source: Interdiscip Cardiovasc Thorac Surg. 2026 Apr 15;41(4):ivag101. doi: 10.1093/icvts/ivag101 (PMC13110008; doi:10.1093/icvts/ivag101)

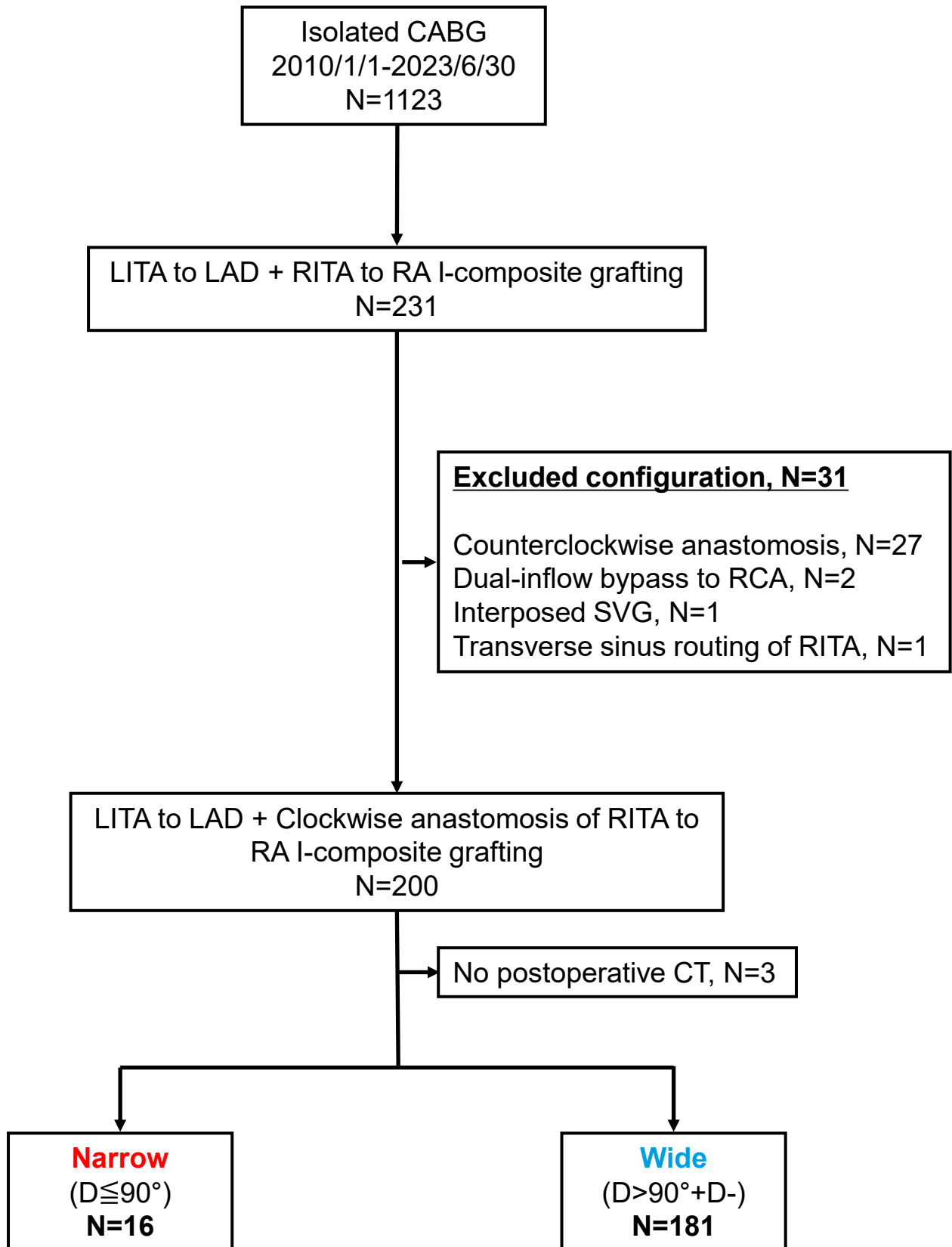

Supplement: ivag101_Supplementary_Data [file ivag101_supplementary_data.zip › Supplementary_Figure_S1.pdf]

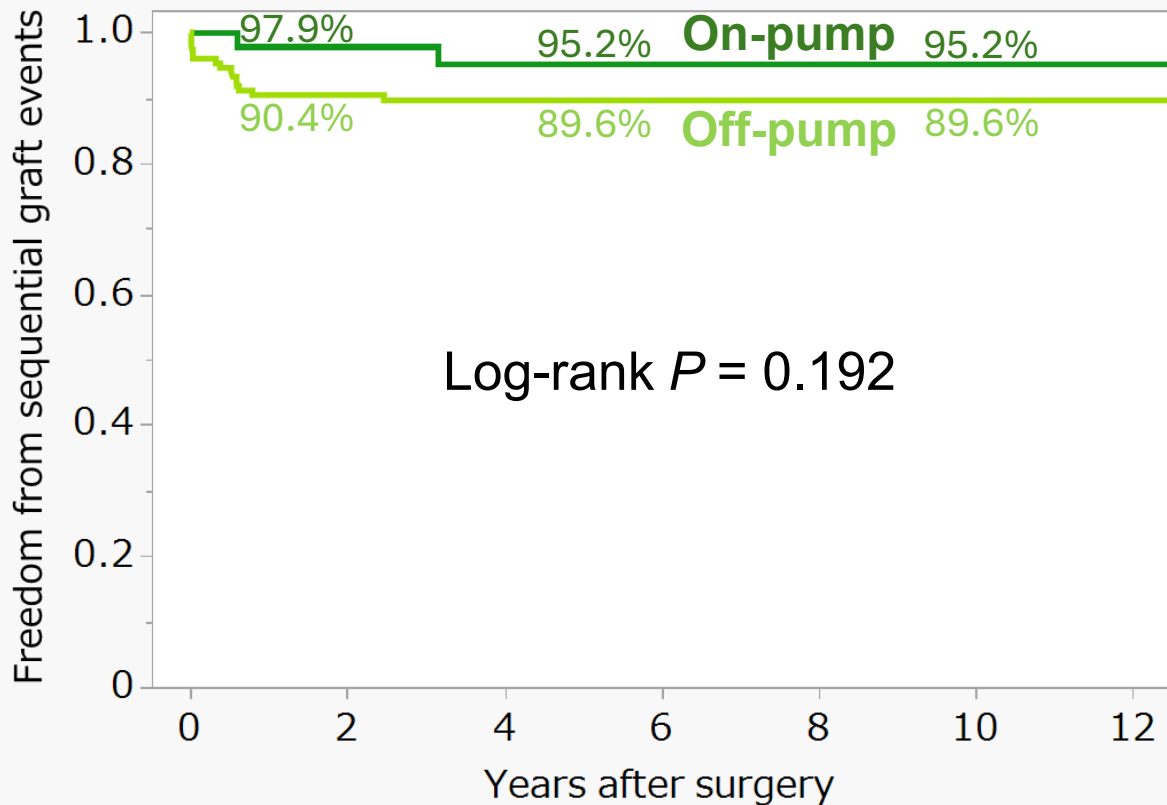

Number at risk

|          |     |     |    |    |    |    |   |
|----------|-----|-----|----|----|----|----|---|
| On-pump  | 48  | 45  | 29 | 17 | 13 | 5  | 2 |
| Off-pump | 149 | 126 | 93 | 73 | 53 | 27 | 4 |

Supplement: ivag101_Supplementary_Data [file ivag101_supplementary_data.zip › Supplementary_Figure_S2.pdf]
